# In-domain versus out-of-domain transfer learning in plankton image classification

**DOI:** 10.1038/s41598-023-37627-7

**Published:** 2023-06-27

**Authors:** Andrea Maracani, Vito Paolo Pastore, Lorenzo Natale, Lorenzo Rosasco, Francesca Odone

**Affiliations:** 1 Istituto Italiano di Tecnologia, Genoa, Italy; 2 MaLGa-DIBRIS, Università degli studi di Genova, Genoa, Italy; 3 CBMM, Massachusetts Institute of Technology, Massachusetts, CA USA

**Keywords:** Image processing, Machine learning

## Abstract

Plankton microorganisms play a huge role in the aquatic food web. Recently, it has been proposed to use plankton as a biosensor, since they can react to even minimal perturbations of the aquatic environment with specific physiological changes, which may lead to alterations in morphology and behavior. Nowadays, the development of high-resolution in-situ automatic acquisition systems allows the research community to obtain a large amount of plankton image data. Fundamental examples are the ZooScan and Woods Hole Oceanographic Institution (WHOI) datasets, comprising up to millions of plankton images. However, obtaining unbiased annotations is expensive both in terms of time and resources, and in-situ acquired datasets generally suffer from severe imbalance, with only a few images available for several species. Transfer learning is a popular solution to these challenges, with ImageNet1K being the most-used source dataset for pre-training. On the other hand, datasets like the ZooScan and the WHOI may represent a valuable opportunity to compare out-of-domain and large-scale plankton in-domain source datasets, in terms of performance for the task at hand.In this paper, we design three transfer learning pipelines for plankton image classification, with the aim of comparing in-domain and out-of-domain transfer learning on three popular benchmark plankton datasets. The general framework consists in fine-tuning a pre-trained model on a plankton target dataset. In the first pipeline, the model is pre-trained from scratch on a large-scale plankton dataset, in the second, it is pre-trained on large-scale natural image datasets (ImageNet1K or ImageNet22K), while in the third, a two-stage fine-tuning is implemented (ImageNet $\rightarrow$ large-scale plankton dataset $\rightarrow$ target plankton dataset). Our results show that an out-of-domain ImageNet22K pre-training outperforms the plankton in-domain ones, with an average boost in test accuracy of around 6%. In the next part of this work, we adopt three ImageNet22k pre-trained Vision Transformers and one ConvNeXt, obtaining results on par (or slightly superior) with the state-of-the-art, corresponding to the usage of CNN models ensembles, with a single model. Finally, we design and test an ensemble of our Vision Transformers and the ConvNeXt, outperforming the state-of-the-art existing works on plankton image classification on the three target datasets. To support scientific community contribution and further research, our implemented code is open-source and available at https://github.com/Malga-Vision/plankton_transfer.

## Introduction

The term *plankton* refers to a large class of drifting aquatic microorganisms. Plankton plays a key role in the aquatic ecosystem, being at the bottom of the marine food chain. Moreover, phytoplankton is estimated to have produced around 50% of the total atmosphere oxygen with fundamental involvement in local and global climate regulation^1^. Plankton community composition is deeply impacted by natural or artificial perturbations of the aquatic environment^2^. Plankton microorganisms can respond to changes in the environment with physiological changes, potentially causing morphological, and behavioral modifications^3^. For these reasons, their usage as biosensors has been proposed: detecting deviations from a computed healthy baseline as indicators of potentially dangerous environmental changes^4,5^.

The development of advanced in-situ high-resolution automatic acquisition systems, e.g., the submersible flow cytometer^6,7^ and the In Situ Ichthyoplankton Imaging System (ISIIS)^8^, is leading to a large amount of available plankton image data. In particular, from 2006 to 2014 the Woods Hole Oceanographic Institution (WHOI) acquired a large-scale dataset comprising millions of plankton images, labeled by experts in the field in 103 categories. Another example is the ZooScan dataset (acquired by means of the homonymous instrument^9^) which includes 1.4 million images labeled into 98 different categories. While there is a growing availability of such data, high-quality unbiased annotations can be costly in terms of both time and resources^10,11^, furthermore there is a pressing need to develop highly accurate algorithms for automatic plankton image classification. To address this challenge, researchers have turned to machine learning solutions, particularly supervised training of Convolutional Neural Networks (CNNs)^12–16^, which have demonstrated superior performance compared to traditional computer vision methods, as highlighted by several studies. CNNs are powerful deep learning architectures commonly used for image classification and object recognition tasks: they consist of multiple convolutional layers that can learn and extract hierarchical representations of input images, allowing the network to identify features of varying complexity^17^.

A widely used approach in plankton image classification is transfer learning, where the weights of a pre-trained CNN architecture on a large general dataset (such as natural images) are fine-tuned with the images and labels of a specific plankton dataset, as proposed by various works^15,18,19^ (the *knowledge* acquired by the model in the pre-trained dataset is *transferred* to make the downstream task, i.e., plankton classification, easier). To achieve state-of-the-art performance in plankton classification, current approaches typically involve fine-tuning multiple CNN models, often six or more, and combining their predictions through ensemble methods to obtain highly accurate results^15,20,21^. These methods typically rely on the widely-used ImageNet1k dataset for pre-training and are evaluated on small-to-medium-sized plankton datasets that have been curated from larger image collections, including WHOI22^22^, Kaggle38^8^ and ZooScan20^9^, where the number following the name denotes the number of classes included in the dataset (see section “Datasets”). ImageNet1K denotes the subset of ImageNet that consists of 1000 natural image classes and was used in the ImageNet Large Scale Visual Recognition Challenge 2012^23^. Conversely, we will refer to the entire dataset, which contains 21, 841 classes, as ImageNet22K.

An important limitation of the aforementioned approaches is that the ensemble requires training of multiple deep neural networks and, also, they should be used concurrently at inference time, impacting the efficiency of the resulting method. Furthermore, only *classical* CNNs are typically considered for plankton classification, and new architectures, designed in recent years, have not been yet fully explored. Additionally, to the best of our knowledge, no study has comprehensively examined the impact of the model pre-training on various large-scale, in-domain plankton datasets versus out-of-domain natural image datasets.

To address these gaps, in this paper, we first design three transfer learning pipelines to compare the effect of in-domain (extended versions of the three cited plankton datasets, comprising up to 1.4 million images) and out-of-domain (ImageNet1K^23^ and ImageNet22K^24^) source datasets when adopting transfer learning on the three plankton benchmark datasets, exploiting a classical CNN model: ResNet50.

Our experiments indicate that using ImageNet22K for pre-training results in a significant improvement of approximately 6% in test accuracy compared to in-domain dataset pre-training alone. This suggests that the complexity and diversity of ImageNet22K provide valuable learning opportunities for effective plankton classification. While representations learned from large-scale in-domain plankton datasets are more specialized to the domain, they may be less discriminative than those learned from ImageNet22K.

In the next part of this work, we adopt more recent and complex architectures trained on ImageNet22K: three types of Vision Transformers (i.e., ViT^25^, Swin^26^ and BEiT^27^) and a modern CNN (i.e. ConvNeXt^28^). Vision Transformers have been introduced in^25^ and, in contrast to CNNs, exploit a self-attention mechanism^29^ to aggregate information from patches of an image, enabling the model to recognize objects by attending to different parts of the image simultaneously. We fine-tune each of the Transformers and the ConvNeXt on our three target plankton datasets and our results show that the BEiT outperforms the ResNet50 model with an average improvement of 2% in terms of test accuracy. Comparing our results with the best ensembling methods, our experiments show that the ImageNet22K pre-trained BEiT Transformer outperforms the state-of-the-art ensembles on Kaggle38 and ZooScan20 and obtains a similar performance on the WHOI22 dataset. Additionally, we investigate whether combining the three Vision Transformers and the ConvNeXt models within an average ensemble architecture could bring further improvement in accuracy. However, the accuracy gain (compared to our best single model) is minimal and counterbalanced by the resulting additional computational complexity. Nevertheless, the ensemble classifier outperforms the state-of-the-art results for all three investigated datasets.

The remainder of the paper is organized as follows: first, we introduce the related works on transfer learning for plankton image classification. Then, we provide details on the datasets (section “Datasets”) and the implemented pipeline (section “Transfer learning pipelines”). Finally, we provide the experiment details (section “Experiment details”), presenting and discussing the obtained results (section “ Results”).

## Related works

In recent years, there has been a growing interest in the computer vision community toward plankton image classification^15^. Starting from 2014, when the Kaggle National Data Science Bowl was organized with the aim to create an accurate classifier for plankton images, machine learning has been extensively applied to the task at hand^5^. The main approaches involve designing and extracting features that are later used to train Random Forest or Support Vector Machine (SVM) classifiers^11,12,22^ or exploit deep learning in the form of Convolutional Neural Networks (CNNs)^11,20,30–35^. Nowadays, large-scale annotated plankton datasets are publicly available (e.g., the ZooScan98^9^ and the WHOI80 datasets^7^). However, plankton datasets are typically imbalanced^36^, and obtaining high-quality annotations is expensive both in terms of time and resources. A popular solution to deal with these challenges involves the usage of a transfer learning framework^15,20,21,34^. In^34^ the authors compare the performance of an SVM classifier trained on features extracted by means of CNNs (i.e., the DeepSea^37^ and the AlexNet^38^) pre-trained on the extended Kaggle plankton dataset^8^ with 30 thousand images and ImageNet1K. The authors find only a slight difference in the performance of AlexNet pre-trained on the Kaggle plankton dataset and ImageNet1K when using it as a features extractor on their in-house dataset. In^15^, the authors adopt an ensemble of different CNN models with three different classification pipelines involving transfer learning, testing them on the same benchmark datasets used in this work. In particular, they compare: (i) a CNN pre-trained on ImageNet1K and fine-tuned on the plankton target datasets; (ii) a two-round fine-tuning procedure, where the ImageNet1K pre-trained model is fine-tuned on a source plankton dataset and further trained on the target plankton datasets. In this work, the source dataset is obtained by fusing the extended version of the Kaggle dataset^8^ (15, 962 images and 83 classes) and a dataset referred to as *Esmeraldo* (11, 005 images and 13 samples). The two-round fine-tuning procedure provides small improvements or degradation of test accuracy, depending on the model and the target dataset, with respect to a direct fine-tuning of the pre-trained model. Moreover, the designed ensemble of CNNs provides a boost in accuracy. In^21^ the authors adopt average and stacking ensembling of six CNN models including a DenseNet^39^ and EfficientNets^40^. All the CNN models are pre-trained on ImageNet1K. Their ensemble of six CNNs outperforms previous state-of-the-art results for the classification of the investigated plankton datasets.

In^35^ the authors compare different transfer learning scenarios using an ImageNet1K pre-trained AlexNet, fine-tuned on the extended Kaggle dataset, an extended version of the WHOI dataset with 53,239 images, and both of them in cascade. Their results show that the ImageNet1K pre-trained CNN is more accurate than the same model pre-trained on a plankton dataset, with the two-stage fine-tuning giving only a slight improvement.

The previously cited works focus on plankton image classification, which is the same task considered in our study. However, it is worth noting that the advantages of pre-training within a transfer learning framework have been investigated in other computer vision tasks applied to plankton, such as specimen detection^41^, where the classification of plankton microorganisms is coupled with localization. Up to our knowledge, no work for specimen detection performs a systematic analysis on the effect of in-domain pre-training for the detection task, with most of the methods based on the fine-tuning of a pre-trained model on the plankton target dataset. In these works, the usage of models pre-trained on out-of-domain source datasets allows compensation for the limited availability of data, that prevents training from scratch. In the context of object detection, deep neural networks are typically pre-trained on Microsoft Common Objects in Context (MS-COCO), which is a popular out-of-domain object detection dataset. In^42^, the authors design a mask region CNN to perform multi-class microorganisms detection. The proposed model is pre-trained on MS-COCO and then fine-tuned on a plankton dataset, achieving good detection performance also on an out-of-domain blood dataset. In^43^, the authors introduce a phytoplankton image dataset, to be used as a candidate source dataset for the specimen detection task. In this work, a Faster R-CNN with an ImageNet pre-trained backbone is fine-tuned on the introduced dataset, showing high detection accuracy. In^44^ an ImageNet pre-trained CNN is exploited to extract features from plankton images in a specimen detection task. The pre-trained features are shown to provide higher accuracy with respect to a set of hand-crafted features, without any fine-tuning on the plankton detection task.

Previous works have not systematically addressed the problem of in-domain versus out-of-domain transfer learning in plankton image analysis. They instead rely on small-scale plankton datasets as sources and typically employ classical CNN models. The ensembles of CNNs designed in these works tend to yield better performance than single models, however, limited insights are provided on the trade-off between increased complexity and computational training/test time and accuracy improvement. To address these gaps, this paper proposes three transfer learning pipelines to systematically evaluate the effectiveness of plankton in-domain and natural images out-of-domain pre-training datasets in a transfer learning framework. We consider source in-domain plankton datasets with up to one million images to allow a fair comparison in terms of the number of images with ImageNet datasets. Finally, we design an ensemble of three Transformers and one ConvNeXt, evaluating its effect in terms of the trade-off between complexity and accuracy gains for the task at hand.

## Methods

### Datasets

**Figure 1:**
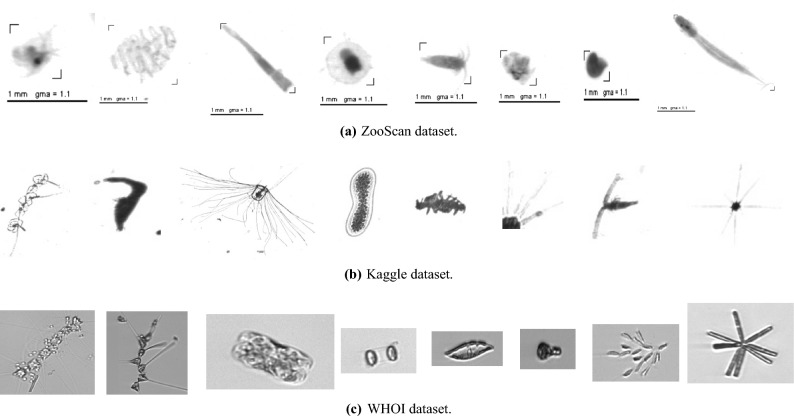
Sample images from seven different classes included in the datasets considered for our analysis.

In this work, we exploit three popular benchmark plankton image datasets. The target datasets are the same used in^12,15,20,21^: (1) WHOI22, (2) Kaggle38; (3) ZooScan20. Each of these datasets is a subset extracted from a corresponding larger collection of annotated images. We consider the correspondent large-scale datasets as in-domain source datasets to pre-train our models when testing the proposed transfer learning pipelines. In the next paragraph, we provide a short description. Figure 1 shows sample images of eight species for each of the three included datasets, while Table 1 provides more details on the number of images and classes included.

#### WHOI dataset

The WHOI dataset^7^ (see Fig. 1c) refers to a public large collection of plankton images acquired by the Woods Hole Oceanographic Institution (WHOI) using automated submersible imaging-in-flow cytometry by means of an Imaging FlowCytobot (IFCB), from 2006 to 2014^6^. The dataset includes 3.4 million images labeled into 103 categories. A subset of the WHOI dataset, introduced in^22^, includes 6, 600 images labeled into 22 categories. This subset is referred to as WHOI22, in our paper. Starting from the whole WHOI dataset, we eliminate all the 22 classes of the WHOI22 and the class labeled as *mix*, obtaining 253, 952 images belonging to 80 different species of plankton. In this paper, we refer to the resulting dataset as WHOI80. We use the WHOI80 as an in-domain source dataset, while the WHOI22 is exploited as a target dataset. The dataset is natively available with a test set, with a number of images equal to the training set.

#### Kaggle dataset

The Kaggle dataset^8^ (see Fig. 1b) refers to a collection of plankton images acquired in the Straits of Florida by means of the In Situ Ichthyoplankton Imaging System (ISIIS), and exploited for the National Data Science Bowl 2015 Kaggle competition. The original labeled version of the dataset includes 30, 336 images belonging to 121 different classes. In^12,15^ the authors use a subset of such dataset, including 14,374 greyscale images labeled into 38 classes. We refer to such a subset as Kaggle38 in the remainder of the paper. Starting from the whole labeled dataset, we remove the samples belonging to the 38 classes of the Kaggle38 subset, obtaining 15,962 plankton images belonging to 83 different categories (as done in^15^). We refer to this version of the dataset as Kaggle83 in the paper. We use the Kaggle83 as an in-domain source dataset and the Kaggle38 as a target dataset to test our transfer learning pipelines. Since no test set is available, we adopt the same test protocol of^12,15^ using a 5-fold cross-validation procedure.

#### ZooScan dataset

The ZooScan dataset^45^ (see Fig. 1a) refers to a large-scale collection of plankton images acquired by means of an instrument named ZooScan^9^. The complete version of the dataset includes 1.4 million images labeled into 98 classes (we refer to this dataset as ZooScan98). A popular benchmark plankton dataset extracted from ZooScan98 is used in many works^12,15^. We refer to such a subset as ZooScan20, it contains 3, 771 greyscale images labeled into 20 classes. We use ZooScan98 as an in-domain source dataset and ZooScan20 as a target dataset to test our transfer learning pipelines. Since no test set is available, we use again the same test protocol of^12,15^ adopting a 5-fold cross-validation procedure.

**Table 1:** Schematic overview of the eight datasets used in this work including number and type of images, number of classes, and role in the transfer learning pipeline.

Dataset	# Images	# Classes	Images type	Role
ImageNet22K	14,197,122	21,841	Natural	Out-domain source
ImageNet1K	1,281,167	1000	Natural	Out-domain source
ZooScan98	1,400,000	98	Plankton	In-domain source
WHOI80	253,952	80	Plankton	In-domain source
Kaggle83	15, 962	83	Plankton	In-domain source
Kaggle38	14, 374	38	Plankton	Target
WHOI22	6600	22	Plankton	Target
ZooScan20	3771	20	Plankton	Target

**Figure 2:**
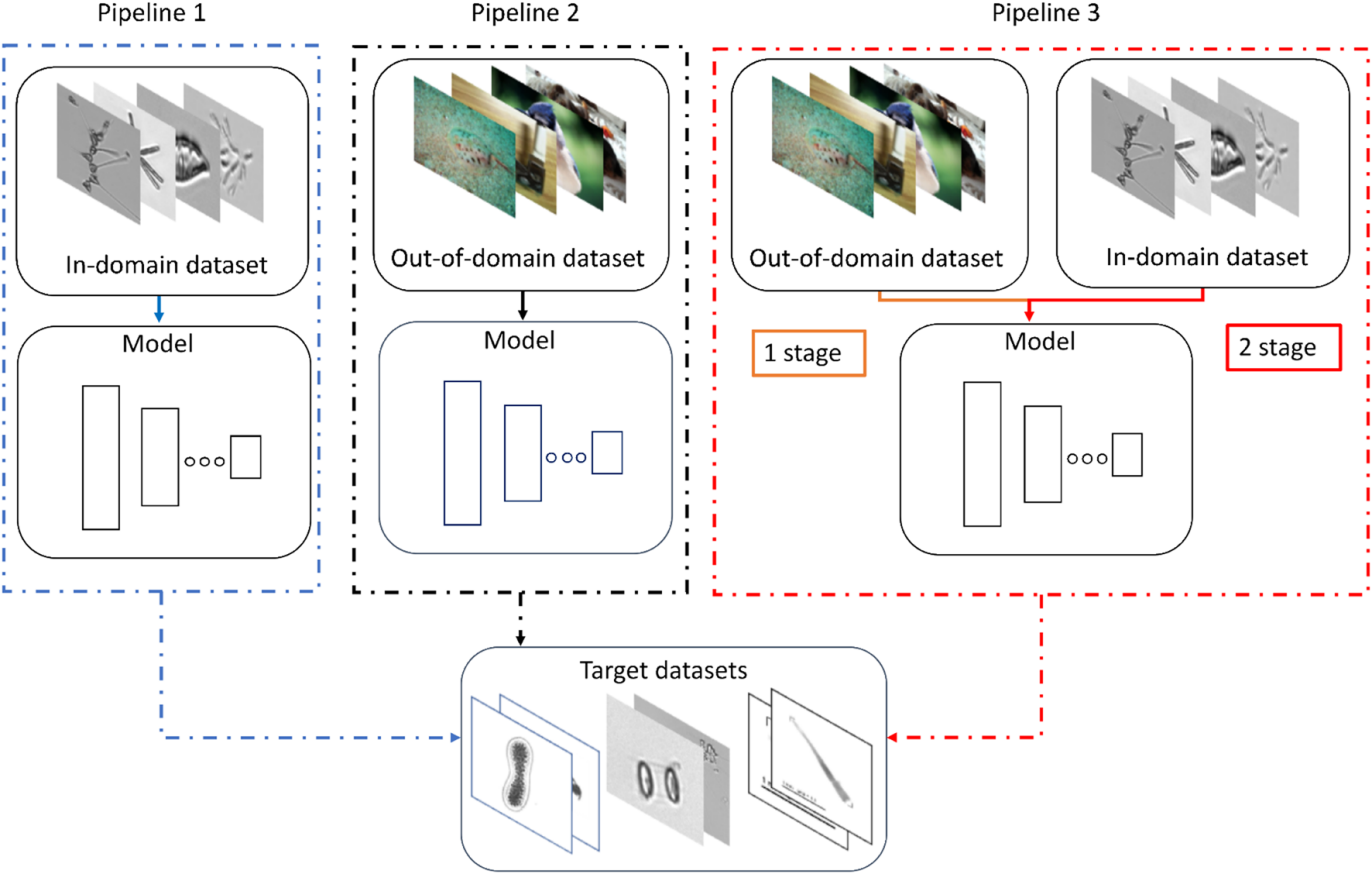
Schematic representation of the three implemented transfer learning pipelines. The dashed blue square corresponds to the first pipeline, where a model is pre-trained from scratch on a large-scale in-domain plankton dataset; the dashed black square identifies the adoption of out-of-domain ImageNet pre-training; the dashed red square represents the two-stage fine-tuning procedure (ImageNet $\rightarrow$ in-domain plankton dataset $\rightarrow$ target dataset).

### Transfer learning pipelines

Figure 2 shows a schematic representation of the pipelines we designed to evaluate the impact of in-domain and out-of-domain transfer learning on plankton image data. In the first transfer learning pipeline (dashed blue square in Fig. 2), we use the extended version of the plankton datasets included in our analysis (see section “Datasets”) as in-domain source datasets to train a ResNet50 model^46^ from scratch. The resulting model is then fine-tuned on each of the three target datasets and evaluated in terms of accuracy and $\text {F}_1 \text { score}$ on the test sets (see section “Evaluation metrics” for further details).

In the second transfer learning pipeline (dashed black square in Fig. 2) we use two popular natural image datasets as out-of-domain source datasets to train a ResNet50 model: ImageNet1K and ImageNet22K. The first is a collection of 1.2 million images belonging to 1000 different classes, while the second includes 14 million images labeled into 21,841 categories^47^. We fine-tune the resulting model on each of the three target datasets and evaluate it in terms of accuracy and $\text {F}_1 \text { score}$ on the test sets. Finally, for the two in-domain plankton datasets with less than one million images (i.e., WHOI80 and Kaggle83), we design a third transfer learning pipeline (dashed red square in Fig. 2) adopting a two-stage fine-tuning procedure, in the attempt to mitigate the effect of the number of images, when comparing to the out-of-domain ImageNet datasets. In particular, we first fine-tune a ResNet50 model pre-trained on ImageNet22K on one plankton in-domain dataset, later performing another stage of fine-tuning on each of the three target datasets.

### Ensemble of transformers and ConvNeXt architectures for plankton image classification

In this work, we first test the designed transfer learning pipelines exploiting a ResNet50 architecture. Then, we consider deeper and more complex architectures, namely Vision Transformers and a ConvNeXt. In particular, we adopt and compare ViT^25^, a hierarchical Transformer (i.e., Swin)^26^, a BEiT Transformer^27^ and ConvNeXt^28^ to accurately classify our target plankton image datasets. All the models are pre-trained on ImageNet22K and fine-tuned on the target datasets. Finally, following the state-of-the-art approaches for plankton image classification, we combine the four models into an ensemble, to evaluate the impact on performance on the target datasets. In particular, we average the output probabilities for each of the models, selecting the output class based on the maximum of the obtained values.

## Results

### Experiment details

#### Image pre-processing

The plankton datasets used in this work include images of different sizes and aspect ratios. An important requirement for the efficient training of a neural network consists in having input images of the same size, allowing them to be batched into tensors for hardware acceleration. Additionally, for Transformer architectures, square input images are desirable as they are divided into a grid of pre-defined square patches during training. Therefore, we follow the resizing strategy employed in previous works^15^: (1) the aspect ratio is maintained by padding the smallest dimension of each image, achieving a square shape; (2) all the images are resized to a fixed size; and (3) a square region is cropped from the resulting image. For ZooScan images, prior to the described pipeline, we automatically remove the artifact represented by the size indication legend. The resize and crop sizes are consistent with the ones used for pre-training each architecture: for ResNet50, images are resized to $256 \times 256$ and then cropped to $224 \times 224$, while for other architectures (ViT, BEiT, Swin, and ConvNeXt), images are resized to $439 \times 439$ and then cropped to $384 \times 384$. During training, the crop is randomly performed across the image as an augmentation technique. During testing, the crop is centered on the image.

#### Training details

Before fine-tuning the model weights, we proceed by substituting the existing fully-connected layers on top of each model with a newly initialized bottleneck. This bottleneck comprises a linear layer with 512 neurons, a normalization layer, and a non-linear activation function. Finally, a linear classification layer is added with the number of output dimensions matching the number of classes. The normalization is a Layer Normalization^48^ (with GELU activation function) or a Batch Normalization^49^ (with ReLU activation function) according to the used backbone (the former for Vision Transformers and ConvNeXt, the latter for ResNet50). We train the final classifier applying Weight Normalization^50^. We use data augmentation based on random horizontal and vertical flips, Stochastic Gradient Descent (SGD)^51^ with Nesterov momentum (0.9) for the optimization, and cross-entropy as loss function. We use regularization with weight decay ($10^{-2}$) and label smoothing (0.1). The initial learning rates are $10^{-3}$ for the pre-trained backbone and $10^{-2}$ for the bottleneck and the classifier. They are decayed with exponential scheduling: at training step *t*, the learning rate is evaluated as the initial learning rate multiplied by $\text {decay}(t) = \left( 1 + \gamma \frac{t}{n}\right) ^{\beta }$ where $\gamma = 10$, $\beta = 0.75$ and *n* is the total number of training steps ($\# {\text {epochs}} \cdot  \# {\text {steps in one epoch}}$). We use 100 epochs with early stopping (training/validation split is 85/15). The batch size is 64, but we split every batch across 4 GPUs (NVIDIA V100 16 GB), exploiting gradient accumulation, when needed. We synchronize batch normalization statistics across GPUs. For our experiments, we used Python (version 3.9.12) with PyTorch library (version 1.11.0) and CUDA 10.2. We imported the architecture implementations from the TIMM library^52^. The ConvNeXt model used in our work is ConvNeXt-XL architecture, while for the Transformers the BEiT-L, ViT-L, and Swin-L implementations are adopted.

#### Evaluation metrics

We evaluate our results by exploiting two common metrics for plankton image classification (as done in^20^): accuracy and $\text {F}_1 \text { score}$, defined as:

1 $$\begin{aligned} \text {Accuracy}:= \frac{\text {Total True Positives}}{\text {Total Instances}} \end{aligned}$$

2 $$\begin{aligned} \text {F}_1\text { score}:=\frac{1}{C} \sum _{i=1}^{C} \text {F}_1\text { score}_i \end{aligned}$$

3 $$\begin{aligned} \text {F}_1 \text { score}_i :=2 \times \frac{\text {Precision}_i \times \text {Recall}_i}{\text {Precision}_i + \text {Recall}_i} \end{aligned}$$

In Eq. (1), *Total True Positives* represents the sum of true positives across all classes, and *Total Instances* represents the total number of images in the test dataset. In Eq. (2), *C* represents the total number of classes, and $\text {F}_1 \text { score}_i$ represents the $\text {F}_1 \text { score}$ corresponding to instances in class *i*. The latter is computed as shown in Eq. (3), where $\text {Precision}_i = \frac{\text {TP}_i}{\text {TP}_i + \text {FP}_i}$ and $\text {Recall}_i = \frac{\text {TP}_i + \text {TN}_i}{\text {TP}_i + \text {FP}_i + \text {FN}_i + \text {TN}_i}$. True Positive ($\text {TP}_i$), True Negative ($\text {TN}_i$), False Negative ($\text {FN}_i$), and False Positive ($\text {FP}_i$) correspond to the element in the confusion matrix of class *i*. In summary, the accuracy metric provides a measure of performance, considering each instance equally important. The $\text {F}_1 \text { score}$ provides a measure of performance considering each class equally important when calculating the average. If a dataset is balanced, with the same number of instances per class, $\text {F}_1 \text { score}$ and accuracy coincide, however, in the case of imbalanced datasets, such as the plankton ones^36^, $\text {F}_1 \text { score}$ may be considered a relevant additional metric in the evaluation of a classification task. Finally, for Kaggle38 and ZooScan20 datasets, the evaluation metrics are averaged among the 5 folds (see section “Datasets”).

### Experiment results

#### In-domain versus out-of-domain transfer learning

We apply the transfer learning pipelines described in section “Transfer learning pipelines” to the three datasets used in this work (see section “Datasets”). The experiments reported in this section, are performed using ResNet50 as a baseline architecture. Table 2 shows the obtained results in terms of accuracy and $\text {F}_1 \text { score}$ evaluated on the test set. It is worth noticing that the three extended versions of the plankton datasets used as source datasets for the in-domain transfer learning pipeline have a different number of images: (1) 15,962 for the Kaggle83; (2) 253,952 for the WHOI80 and (3) 1.4 million for the ZooScan98. As a comparison, ImageNet22K has 14 million images belonging to 21,841 classes. ImageNet1K is a subset of ImageNet22K with 1.2 million images belonging to 1000 classes (with a size comparable to the ZooScan98 plankton dataset). As we can see in Table 2, ImageNet22K pre-training leads to the most accurate model for the WHOI22 and the Kaggle target datasets both in terms of accuracy and $\text {F}_1 \text { score}$. ImageNet22K also leads to the best $\text {F}_1 \text { score}$ for the ZooScan dataset, while there is a slight improvement when using a two-stage fine-tuning involving the WHOI dataset (+  0.004%) w.r.t. the test accuracy, on this dataset. Moreover, if we consider only the in-domain transfer learning pipeline, it is possible to notice that the ZooScan98 dataset leads to the best results for both the WHOI22 and the Kaggle dataset, with an average improvement of around 3.6% w.r.t. pre-training on the other two extended plankton datasets. We do not use ZooScan98 as a source dataset for the fine-tuning on ZooScan20, because it contains all the images and the classes included in the target dataset. In fact, differently from WHOI80 and Kaggle83 extended dataset, we do not remove the classes in common with the target dataset for ZooScan98, because we are interested in considering a dataset with a size comparable to ImageNet1K, in order to fairly compare one in-domain plankton dataset to the external natural images dataset removing the number of images as potential influencing parameter. Our findings suggest that using in-domain plankton datasets as sources in transfer learning frameworks, has a limited or no effect on the accuracy of tested models, while the number of classes and images in a source dataset are important factors that contribute to the quality of a pre-training dataset.

**Table 2:** Performance comparison (accuracy and $\text {F}_1 \text { score}$) of ResNet50 using the proposed transfer learning pipelines across the three benchmark datasets.

Target dataset $\rightarrow$	WHOI22		Kaggle38		ZooScan20	
$\downarrow$ Source dataset(s)	Accuracy	$\text {F}_1 \text { score}$	Accuracy	$\text {F}_1 \text { score}$	Accuracy	$\text {F}_1 \text { score}$
WHOI80	0.878	0.878	0.876	0.831	0.826	0.837
Kaggle83	0.862	0.862	0.878	0.834	0.847	0.863
ZooScan98	0.912	0.912	0.914	0.884	–	–
ImageNet22K	**0.946**	**0.946**	**0.930**	**0.909**	0.887	**0.899**
ImageNet1K	0.939	0.939	0.921	0.895	0.851	0.868
ImageNet22K $\rightarrow$ WHOI80	**0.946**	**0.946**	0.924	0.905	**0.891**	0.898
ImageNet22K $\rightarrow$ Kaggle83	0.938	0.938	0.929	0.907	0.877	0.896

The best results are highlighted in bold, second best results are underlined.

#### Exploiting the pre-training on ImageNet22K: transformers and ConvNeXt for plankton classification

The out-of-domain natural image dataset ImageNet22K corresponds to the best source dataset when pre-training a ResNet50 in our experiments, in terms of test accuracy. Having this in mind, we investigate the performance of more complex architectures that could benefit even more from an ImageNet22k pre-training. In particular, we consider three different Transformers: ViT^25^, the hierarchical Swin Transformer^26^ (Swin) and BEiT^27^. We also include a modern CNN, i.e., ConvNeXt^28^, in our analysis. Table 3 shows the performance of each of these models on the three plankton benchmark datasets. In our experiments, the three Transformers and the ConvNeXt model are pre-trained on ImageNet22K. As we can see, BEiT Transformer shows the highest performance both in terms of test accuracy and $\text {F}_1 \text { score}$, with an average improvement of 2% with respect to the ResNet50 model pre-trained on ImageNet22K (see Table 2). As a benchmark, we compare our results with four recent state-of-the-art works on plankton image classification^12,15,20,21^. Table 4 summarizes state-of-the-art results on the three investigated target plankton datasets. Excluding^12^, the state-of-the-art benchmark results are obtained by ensembling several ImageNet1K pre-trained CNN models (six CNNs in^21^, eleven in^15^). As we can see in Table 3, our single BEiT model outperforms the state-of-the-art results for the Kaggle and the ZooScan dataset, with performance comparable to^21^ on the WHOI22 dataset, where an ensemble of six CNN models is used.

Nonetheless, inspired by previous state-of-the-art results in plankton image classification, we design an average ensemble of our ImageNet22K pre-trained Transformers and ConvNeXt (see section “Ensemble of Transformers and ConvNeXt architectures for plankton image classification” for further details) to assess the effect on performance with respect to the three target datasets. As we can see in Table 4, the resulting ensemble model provides a minimal effect on accuracy, with an average increase of around $0.6\%$ with respect to our best performing Transformer (i.e., BEiT).

**Table 3:** Performance comparison (accuracy and $\text {F}_1 \text { score}$) of Vision Transformers, ConvNeXt, and ResNet50 (as baseline) pre-trained on ImageNet22K across the three benchmark datasets.

Dataset $\rightarrow$	WHOI22		Kaggle38		ZooScan20	
$\downarrow$ Model	Accuracy	$\text {F}_1 \text { score}$	Accuracy	$\text {F}_1 \text { score}$	Accuracy	$\text {F}_1 \text { score}$
ResNet50	0.946	0.946	0.930	0.909	0.887	0.899
BEiT	**0.961**	**0.961**	**0.951**	**0.942**	**0.914**	**0.931**
Swin	0.960	0.960	0.947	0.932	0.904	0.917
ViT	0.959	0.959	0.948	0.933	0.908	0.918
ConvNeXt	0.957	0.957	0.949	0.932	0.904	0.911

The best results are highlighted in bold, second best results are underlined.

**Table 4:** Performance comparison (accuracy and $\text {F}_1 \text { score}$) of our best single model (BEiT) and our ensemble of 4 models with state-of-the-art approaches on three investigated plankton datasets.

Dataset $\rightarrow$	WHOI22		Kaggle38		ZooScan20	
$\downarrow$ Method	Accuracy	$\text {F}_1 \text { score}$	Accuracy	$\text {F}_1 \text { score}$	Accuracy	$\text {F}_1 \text { score}$
Best 6 average^21^	0.961	0.961	0.947	0.937	0.898	0.915
Best 6 stack^21^	0.958	0.958	0.943	0.934	0.891	0.911
SFFS^15^	0.958	0.958	0.942	0.927	0.885	0.900
WS^15^	0.958	0.958	0.942	0.927	0.888	0.902
Fus 2R + Fus 1R^20^	–	0.953	–	0.926	–	0.897
Fus PR+ Fus 2R +Fus 1R^20^	–	0.953	–	0.926	–	0.896
NLMKL^12^	–	0.900	–	0.846	–	0.894
BEiT (**ours**)	0.961	0.961	0.951	0.942	0.914	0.931
Ensemble (4 models, **ours**)	**0.966**	**0.966**	**0.955**	**0.945**	**0.925**	**0.937**

The best results are highlighted in bold and the second best results are underlined.

However, the minimal increase in accuracy is counterbalanced by a significant increase in time and resources needed for training and inference. Table 5 reports an indication of training and inference time, as the number of images that can be processed per second, by the different architectures considered in our study (and by the ensemble of the 4 architectures) on a single NVIDIA V100 GPU. These numbers depend on the specific hardware and implementation. However, they highlight the difference, in terms of efficiency, among the architectures, and the increase in time needed for computation when ensembling the four models. Thus, the trade-off between complexity and accuracy gain should be carefully evaluated, depending on the specific application (e.g., real-time or post-acquisition analysis).

**Table 5:** The average number of images processed by our models in one second at training and inference time.

Model	BEiT	ViT	SWIN	ConvNeXt	Ensemble
Training (imgs/s) $\uparrow$	20.32	21.72	32.57	13.16	4.95
Inference (imgs/s) $\uparrow$	65.68	70.26	102.70	52.88	17.21

The values have been evaluated based on 1000 iterations. The higher the value, the faster the processing time.

## Conclusion

In this work, we compare in-domain and out-of-domain transfer learning approaches for plankton image classification. We design three different transfer learning pipelines using three large-scale in-domain source plankton datasets (i.e., WHOI80, Kaggle83, and ZooScan98) and two out-of-domain natural image datasets (i.e., ImageNet1K and ImageNet22K).

The general framework consists in fine-tuning a pre-trained model on three target plankton datasets (i.e., WHOI22, ZooScan20, and Kaggle38). In the first pipeline, we train a model from scratch on an in-domain plankton dataset. In the second pipeline, we adopt an ImageNet1K or ImageNet22K pre-trained model, while in the third, we implement a two-stage fine-tuning procedure, fine-tuning an ImageNet pre-trained model on an in-domain source plankton dataset.

Regarding the first pipeline, we exploit three in-domain source datasets with different numbers of images and classes (see section “Datasets”). Our experiments show that the ZooScan98 dataset with 1.4 million images and 98 classes provides the best performance when used as a source dataset, with an average improvement of 3.6% compared to the pre-training with the other two in-domain datasets.

From the second pipeline, we obtain that ImageNet22K provides better performance compared to ImageNet1K, with an average improvement of 4%. These results suggest that there is no benefit in using a large-scale in-domain plankton dataset as a source dataset for transfer learning compared to the out-of-domain ImageNet. Moreover, little or no benefit is obtained when adopting a two-stage fine-tuning procedure. It is worth noticing that ZooScan98 has a higher number of images than ImageNet1K, but leads to lower performance when used as a source dataset. These results may indicate that the number of images and classes are key factors for a pre-training dataset in a plankton image classification task. It is worth noticing that, despite acquiring and annotating large-scale plankton datasets (as ZooScan98) is expensive in terms of time and resources, our experiments show that the usage of in-domain pre-training datasets provides no benefit with respect to ImageNet.

In the next experiments, we adopt current state-of-the-art architectures (ViT, Swin, BEiT, and ConvNeXt, pre-trained on ImageNet22K). The pre-trained models are fine-tuned on the target plankton datasets, providing an average accuracy boost of 2% with respect to the ResNet50 model pre-trained on ImageNet22K. As a benchmark, we compare the obtained results to recent state-of-the-art plankton image classification works, where ensembles of CNN models (up to 11) are used for the task at hand. Our results show that our single BEiT model achieves better performance than state-of-the-art on the Kaggle and the ZooScan datasets, with similar performance to^21^ for the WHOI dataset. Following the current trend in plankton image classification, we further design and test an average ensemble of the three transformers and the ConvNeXt. The designed ensemble brings a slight improvement with respect to the ImageNet-22K pre-trained BEiT. However, it should be noted that such a boost in accuracy ($0.6\%$ on average) is counterbalanced by a significant increase in the computational resources and the training/inference time for the final model.

## Data Availability

All the code needed to reproduce our results is open-source and available at https://github.com/Malga-Vision/plankton_transfer. The target plankton datasets are available at: Kaggle38^8^; ZooScan20^45^ and WHOI22^22^. The code for downloading the extended version is included in the shared repository.
